# Large-Scale Expansion of Human Umbilical Cord-Derived Mesenchymal Stem Cells in a Stirred Suspension Bioreactor Enabled by Computational Fluid Dynamics Modeling

**DOI:** 10.3390/bioengineering9070274

**Published:** 2022-06-23

**Authors:** Junhong Zhang, Yan Peng, Meijin Guo, Chao Li

**Affiliations:** State Key Laboratory of Bioreactor Engineering, East China University of Science and Technology, 130 Meilong Rd., Shanghai 200237, China; y10180350@mail.ecust.edu.cn (J.Z.); 010130181@mail.ecust.edu.cn (Y.P.)

**Keywords:** UC-mesenchymal stem cells, scale-up expansion, computational fluid dynamics, microcarrier-based bioreactors, three-dimensional suspension culture

## Abstract

Human umbilical cord-derived mesenchymal stem cells (hUCMSCs) hold great potential to generate novel and curative cell therapy products. However, the current large-scale cultivation of hUCMSCs is based on empirical geometry-dependent methods, limiting the generation of high-quantity and high-quality hUCMSCs for clinical therapy. Herein, we develop a novel scale-up strategy based on computational fluid dynamics (CFD) to effectively expand the hUCMSCs in a 3D tank bioreactor. Using a standardized hUCMSCs line on microcarriers, we successfully translated and expanded the hUCMSCs from a 200 mL spinner flask to a 1.5 L computer-controlled bioreactor by matching the shear environment and suspending the microcarrier. Experimental results revealed that the batch-cultured hUCMSCs in bioreactors with an agitation speed of 40 rpm shared a more favorable growth and physiological state, similar to that run at 45 rpm in a 200 mL spinner flask, showing comparability in both culture systems. Notably, the maximum cell density reached up to 27.3 × 10^5^ cells/mL in fed-batch culture, 2.9 folds of that of batch culture and 20.2 times of seeding cells. As such, efficient process optimization and scale-up expansion of hUCMSCs were achieved in the microcarrier-based bioreactor system by the developed CFD simulation strategy, which provided an alternative toolbox to generate massive and standardized curative cell therapy products.

## 1. Introduction

Human umbilical cord-derived mesenchymal stem cells (hUCMSCs) are a class of pluripotent stem cells derived from the mesoderm and ectoderm in early development [1,2]. Under certain conditions, hUCMSCs with extremely low immunogenicity could differentiate into the targeted functional cells [3,4,5,6] or secrete the related functional cytokines for an immune response [7,8], exhibiting the potential for wide range of inflammation disease treatment [9]. Although hUCMSCs were used in a number of clinical trials to investigate their potential in immune regulation, tissue repair and organ regeneration, the development and production of hUCMSCs for cell-replacement therapies have only been performed on a small scale that was not suitable for the generation of sufficient cells required for most therapies [10,11]. For instance, it is estimated that 10^9^ cells would be required per patient in heart failure, and the market for heart failure potentially requires thousands of doses a year, requiring 10^11^–10^14^ cells annually [12,13]. Thus, it is of great urgency to develop a robust platform for the high-quantity and high-quality expansion of hUCMSCs [14,15].

Three-dimensional stirred tank bioreactors are proven to be a robust strategy for the large-scale culture of stem cells due to their accommodation of various volumes [16,17]. A suitable shear flow field during the culture process can not only provide a sufficient two-phase contact interface for mass transfer but also exert no damage to stem cells [18,19,20]. However, the key spatial and temporal information of the parameters, such as shear force and flow field characteristics, are empirically optimized and geometry-dependent for application-specific purposes [21,22,23,24], which greatly limits the understanding of how fluid shear stresses and other physical forces affect stem cell behaviors, and subsequently hinder the rational design of scalable cell bioreactors when utilizing fluid shear stress as a key input parameter [25,26].

Recently, computational fluid dynamics (CFD) has played a strong supporting role in calculating the hydrodynamic forces in modeling [27,28,29,30,31], where the geometry differences between small and large-scale vessels could be comprehensively considered to predict the favorable flow field characteristics in different size and/or shape vessels [31,32,33,34,35]. Thus, we conjectured that the high-quantity and high-quality expansion of hUCMSCs grown in a 3D stirred tank bioreactor could be achieved using CFD simulation.

Herein, we adopted the computational fluid dynamics (CFD) method to obtain optimized flow field characteristics in a 200 mL spinner-flask, where growth and substrate metabolism of the batch-cultured hUCMSCs as well as the expression of characteristic markers were comprehensively evaluated. Using association analysis between the shear-related parameters and the cell growth states, we predicted the volume average shear force and the growth of hUCMSCs in a three-dimensional suspension culture system. As an example, we developed a 200 mL spinner-flask scale protocol for hUCMSCs culture and reproduced the protocol in a 1.5 L computer-controlled bioreactor based on the scale-up criteria of matching similar shear environment and ensuring the suspension of microcarries. Notably, under the optimized conditions, the maximum cell density of 27.3 × 10^5^ cells/mL could be achieved by fed-batch culture, 2.9-folds of that of batch culture and 20.2 times of seeding cells. We demonstrated the CFD simulation strategy would provide a reference for the optimization and large-scale expansion of the stem cell culture process, which will open up a broader experimental research prospect and potential industrial application for clinical therapy.

## 2. Materials and Methods

### 2.1. Culture of Human Umbilical Cord-Derived MSCs

Culture of human umbilical cord-derived MSCs. All experiments were approved by the Ethics Committee of the National Academy of science and implemented according to the guidelines established by the Ministry of Health of the People’s Republic of China. Human umbilical cords were obtained from healthy full-term caesarian section births according to the previously reported protocols [36] and aseptically stored at 4 °C PBS containing 100 U/mL penicillin/streptomycin solution (Life Technologies, Grand Island, NY, USA). After rinsing and successfully removing blood vessels and perivascular tissues, the remaining cords were cut into 1.0–2.0 mm^3^ tissue blocks and transferred to a sterile container. Then, the MSCs were isolated by enzymatic digestion by adding 40 mL of 0.02% type II collagenase (298 U/mg; Sigma-Aldrich, St. Louis, MO, USA) and 0.05% hyaluronidase, followed by incubation on a shaker for 12 h (37 °C, 5% CO_2_ and 95% air). After that, an α-MEM growth medium with 10% fetal bovine serum was added to terminate the digestion process; then, a 200-mesh cell sieve was used to remove undigested tissue and collect the filtrate. Finally, the filtrate containing MSCs was centrifuged at 1000 rpm for 5 min to obtain the desired Passage 0 MSCs derived from human umbilical cords. hUCMSCs on the third passage (P3) was adopted for the subsequent experiments.

### 2.2. Spinner Flask and 1.5 L Computer-Controlled Bioreactor Setup for hUCMSCs Expansion

Next, 200 mL spinner flasks (Bellco Biotechnology, El Cerrito, CA, USA) with a flat bottom and large paddle impeller (D = 50 mm) in the bottom of the vessel were first used. Commercial microcarriers (Cytodex3, 0.24 g, Sigma-Aldrich, St. Louis, MO, USA) in 100 mL α-MEM with 10% FBS were supplied. Then, hUCMSCs were inoculated in the spinner flask with a density of 1.35 × 10^5^ cells/mL and cultured at 37 °C for 7 days under a 5% CO_2_ atmosphere. Stirring speed was, respectively, set as 35, 45 and 55 rpm/min. For the scale-up culture of hUCMSCs, a 1.5 L tank bioreactor (BioStar 1.5c, Shanghai Guoqiang Bioengineering Equipment Co., Ltd., Shanghai, China) equipped with dissolved oxygen (DO) probe (Mettler Toledo, Greifensee, Switzerland) and a pH probe (Mettler Toledo, Greifensee, Switzerland) was designed. Both the DO (40%) and pH (pH = 7.2) were computer-controlled. Different from the spinner flask, the bioreactor platforms have different size and shape vessels and impeller configurations. For example, the bioreactor has an elliptical bottom and is agitated by an overhead motor with a 45° elephant ear impeller (D = 60 mm). hUCMSCs were seeded on Cytodex3 microcarriers with a density of 1.35 × 10^5^ cells/mL and cultured at 37 °C for 7 days with different agitation speeds. BioStar 1.5c were operated at working volumes of 600 mL. The pH probe was calibrated using a two-point calibration method with standard pH solutions. For the DO probe calibration, the vessels were filled with α-MEM and 10% FBS (600 mL).

### 2.3. Growth Kinetics Evaluation

The morphology of hUCMSCs was directly visualized under an Invitrogen EVOS FL Auto Cell Imaging System. All the cell viabilities and densities were determined using an automatic counting cell analyzer (CountStar^®^MD F4, Shanghai Ruiyu, Shanghai, China). Namely, the suspension system (1.0 mL) obtained from the spinner flask or BioStar 1.5c stirred bioreactor (*n* = 3 per group, per time point, such as days 1, 2, 3, 4, 5, 6 and 7) were centrifuged, then the microcarriers were further incubated in 0.25% Pancreatin (Sigma-Aldrich, St. Louis, MO, USA) at 37 °C for 5 min to dissociate the cell aggregates. Wash medium (α-MEM) was added in a volume equal to five folds of the collagenase re-suspension volume. Then, the cells were obtained by removing the microcarriers with a 200-mesh cell sieve.

### 2.4. Substrate and Metabolic Intermediate Analysis

The supernatant (3 mL) were obtained from the samples, and the concentration of glucose, glutamine, lactate and ammonia in the media (*n* = 3 per group, per time point) was analyzed using Roche Cedex Bio Analyzer (Roche, Basel, Switzerland). Average specific uptake and production rates were determined by evaluating the change in nutrient concentration over time on a per-cell basis, based on the number of viable cells (*n* = 3 per group).

### 2.5. Assessment of the Characteristic Biomarkers of hUVMSCs via Flow Cytometry

The harvested hUCMSCs were stained with fluorescent-labeled monoclonal antibodies, including positive markers (FITC CD90, PE CD105 and PC7 CD73) and negative ones (APC CD14, CD19, CD34, CD45 and HLA-DR). All the markers were purchased from BD Biosciences (Franklin Lakes, NJ, USA). IgG was used as an isotype control and cultured under the same condition. The stained cells were sorted into defined populations using fluorescent-activated cell sorting (FACS, Beckman Coulter, Chino, CA, USA) to produce a pure population with the appropriate cell marker profile.

### 2.6. CFD Modeling

**Figure 1:**
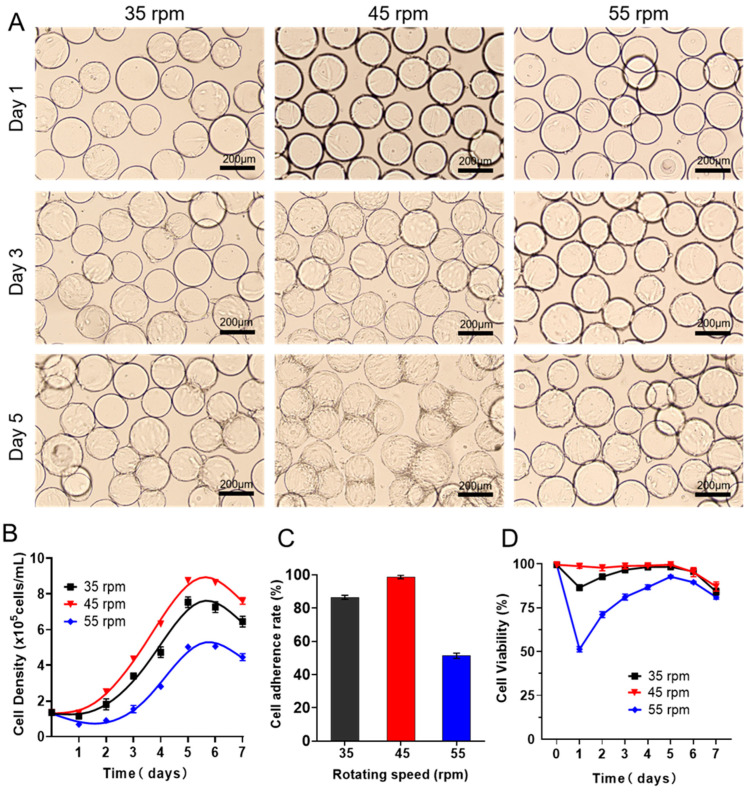
Expansion of batch-cultured hUCMSCs in 3D spinner flask. (**A**) Representative images of hUCMSCs on Cytodex3 cultured in 3D spinner flask with different rotating speeds (35 rpm, 45 rpm, 55 rpm). (**B**) Cell density changes when culturing the P3 hUCMSCs for 1–7 days. (**C**) Cell adherence rate on day 1 after seeding. (**D**) Cell viability during expansion of hUCMSCs. Data expressed as the mean ± standard deviation (*n* = 3 per group, per time point).

In this study, the hydrodynamics of stirred tank bioreactors were investigated at two different scales (200 mL spinner flask and BioStar 1.5c, work volume was, respectively, 100 and 600 mL). The spinner flask (Bellco Biotechnology, El Cerrito, CA, USA) was used as a baseline to scale-up the expansion of hUCMSCs to the 1.5 L bioreactor (BioStar 1.5c, Shanghai Guoqiang Bioengineering Equipment Co., Ltd., Shanghai, China). A schematic presentation of the stirred tanks is provided in Figure 1. Both the bioreactor platforms have a cylindrical vessel but different vessel and impeller configurations. A total of 10 models were run by testing the two different vessels at five different speeds (10, 20, 30, 40 and 50 rpm for the flask and 30, 40, 50, 60 and 70 rpm for Bioreactor 1.5c). At the completion of the simulations, postprocessing was performed to determine the volume average shear strain rate (SSR) for both the spinner flask and BioStar 1.5c. The SSR was then plotted as a function of impeller speed to generate equations that were then used to determine the impeller speed required in the BioStar 1.5c to maintain the same values in the spinner flask.

As the aeration rate during the cultivation of hUCMSCs is less than 0.05 vvm, the effect of aeration on the power consumption of agitation (which is related to the shear rate directly) is much less than 5% [34]. Therefore, the effects of aeration on the flow field and the subsequent shear environment could be ignored, and CFD simulations were performed in a two-phase system with liquid at the lower part of the tank and gas at the upper part of the tank. In this study, the volume of fluid (VOF) model was used to track the free surface formed in the tank. Briefly, the VOF approach is a numerical technique based on solving the transport equation of the fraction function, which is defined as the integral of the fluid’s characteristic functions in the control volume. This equation can be expressed as follows:

(1) $$\frac{\partial \gamma }{\partial t}+(\nu \cdot \nabla )\gamma =0$$

where *γ* is the fraction function, *γ* = 0 where there is no tracer fluid in the element, *γ* =1 is where there is full tracer fluid in an element, 0 < *γ* < 1 is where there are multiple phases in an element. **ν** is the velocity field.

In the VOF model, the liquid and gas velocities are assumed to equilibrate over short distances, and a homogeneous momentum conservation equation is solved as the velocity field for both the liquid and gas phases. Thus:

(2) $$\frac{\partial \rho }{\partial t}\nabla \cdot (\rho \nu )=0$$

(3) $$\frac{\partial }{\partial t}\left(\rho \nu \right)+\nabla \cdot \left(\rho \nu \nu \right)=-\nabla p+\nabla \cdot \left[\mu \left(\nabla \nu +\nabla \nu^{T}\right)\right]+\rho g+F$$

where *p* is pressure. The density and viscosity used above are defined as:

(4) $$\rho =\gamma \rho_{a}+(1-\gamma )\rho_{b}$$

(5) $$\mu =\gamma \mu_{a}+(1-\gamma )\mu_{b}$$

where subscribes *a* and *b* denote the different fluids.

In Equation (3), **F** means the surface tension acting on the gas-liquid interface, modeling as a body force, described as follows:

(6) F=σκ(x)γ

(7) $$\kappa =\frac{1}{\left|n\right|}\left[\left(\frac{n}{\left|n\right|}\cdot \nabla \right)\left|n\right|-\nabla \cdot n\right]$$

where *σ* is the surface tension coefficient, and *κ* is the surface curvature. **n** stands for the unit normal vector of any point in the interface, given by:

(8) n=∇γ.

The turbulence was modeled by the RNG *k*-*ε* model, which is based on renormalization group analysis of the Navier–Stokes equations. The transport equations for turbulence generation and dissipation are the same as those for the standard *k*-*ε* model, but the model constants differ. Thus:

(9) $$\frac{\partial \left(\rho k\right)}{\partial t}+\nabla \cdot \left(\rho \nu k\right)=\nabla \cdot \left[\left(\mu +\frac{\mu_{t}}{\sigma_{k}}\right)\nabla k\right]+P_{k}+P_{kb}^{}-\rho \varepsilon$$

(10) $$\frac{\partial \left(\rho \varepsilon \right)}{\partial t}+\nabla \cdot \left(\rho \nu \varepsilon \right)=\nabla \cdot \left[\left(\mu +\frac{\mu_{t}}{\sigma_{\varepsilon RNG}}\right)\nabla \varepsilon \right]+\frac{\varepsilon }{k}\left(C_{\varepsilon 1RNG}\left(P_{k}+P_{\varepsilon b}\right)-C_{\varepsilon 2RNG}\rho \varepsilon \right)$$

where *k* is turbulent kinetic energy and *ε* is energy dissipating rate. *σ_k_*, *σ_εRNG_* and *C_ε_*_2*RNG*_ are constants and equal to 1.0, 0.7179 and 1.68, respectively. *P_kb_* and *P_εb_* represent the influence of the buoyancy forces. *μ_t_* is the turbulence viscosity. *P_k_* is the turbulence production due to viscous forces, which is modeled using:

(11) $$P_{k}=\mu_{t}\nabla \nu \cdot \left(\nabla \nu +\nabla \nu^{T}\right)-\frac{2}{3}\nabla \cdot \nu \left(3\mu_{t}\nabla \cdot \nu +\rho k\right).$$

*C_ε_*_1*RNG*_ in Equation (10) is the RNG *k*-*ε* turbulence model coefficient, described as:

(12) $$C_{\varepsilon 1RNG}=1.42-f_{\eta }$$

(13) $$f_{\eta }=\frac{\eta \left(1-\frac{\eta }{4.38}\right)}{\left(1+\beta_{RNG}\eta^{3}\right)}$$

(14) $$\eta =\sqrt{\frac{P_{k}^{}}{\rho C_{\mu RNG}\varepsilon }}.$$

In Equations (13) and (14), *β_RNG_* and *C_μRNG_* are constants and equal to 0.012 and 0.085, respectively.

The shear strain rate (SSR, also short for shear rate) can be calculated based on the velocity components *u_x_*, *u_y_*, and *u_z_*, using the following equation:

(15) $$\mathrm{SSR}={\left[2\left\{{\left(\frac{\partial u_{x}}{\partial x}\right)}^{2}+{\left(\frac{\partial u_{y}}{\partial y}\right)}^{2}+{\left(\frac{\partial u_{z}}{\partial z}\right)}^{2}\right\}+{\left(\frac{\partial u_{x}}{\partial y}+\frac{\partial u_{y}}{\partial x}\right)}^{2}+{\left(\frac{\partial u_{x}}{\partial z}+\frac{\partial u_{z}}{\partial x}\right)}^{2}+{\left(\frac{\partial u_{y}}{\partial z}+\frac{\partial u_{z}}{\partial y}\right)}^{2}\right]}^{\frac{1}{2}}.$$

### 2.7. Numerical Strategies

The liquid in the tank was modeled as a Newtonian fluid with the nominal properties of water, and the gas phase was modeled as air under normal conditions. Simulations were performed under various agitation conditions (10–70 rpm). Virtual geometry models were created of the two bioreactors using the computer-aided design (CAD) software Creo 5.0. Fluid flow was simulated with the commercial software ANSYS CFX 15.0 (ANSYS Inc., Canonsburg, PA, USA). An unstructured tetrahedral mesh was generated for the bioreactor using a commercial grid generation software ANSYS ICEM CFD 15.0 (ANSYS Inc., USA), and grids were refined near the impeller zone, resulting in about 1,500,000 elements (with the smallest size of 0.2 mm and the biggest size of 1 mm) in total. The liquid surface was modeled as a free-surface boundary condition. A multi-reference frame (MRF) was used to model the rotation of the impeller. A semi-implicit method for the pressure-linked equations (SIMPLE) algorithm for numerically solving the realizable k-epsilon Navier–Stokes equation was used. All equations were discretized using a high-resolution scheme. For the iterated residuals (10^−4^), impeller torque was monitored to ensure a converged result (it was considered as converged when the residual was less than 10^−4^ and both torque and air volume fraction were kept at a steady-state value). The solver ran on a Sugon cluster platform (Sugon Co., Ltd., Beijing, China) with 96 processors.

### 2.8. Suspension Determination

In total, 1.8 g of microcarrier and 600 mL of the medium were added to the bioreactor. The collected samples were diluted (*n* = 3) and placed in 96-well plates for counting analysis by microscopy.

### 2.9. Statistical Analysis

All data are representative of experiments performed in three independent samples. Data were expressed as mean value ± standard deviation (SD). The statistical significance of the mean values was compared by one-way ANOVA (analysis of variance) and Student’s *t*-test using GraphPad Prism version 8.0.2 software (GraphPad Software Inc., San Diego, CA, USA). *p*-Values < 0.05 were considered to indicate a significant difference.

## 3. Results and Discussions

### 3.1. Expanding hUCMSCs in a 200 mL Spinner Flask in Batch Culture

As a key input parameter, the fluid shear force could not only provide sufficient two-phase contact interfaces for material and energy diffusion but probably damage the cells and affect the growth rate. Thus, it is very necessary to figure out the suitable fluid shear velocity before scale-up culture. To this end, we firstly cultured passage 3 hUCMSCs in a 200 mL spinner flasks equipped with a large paddle impeller (100 mL working volume, 1.35 × 10^5^ cells/mL), where commercial Cytotex3 was used as microcarriers for suspension culture. As shown in Figure 1A, a small shear force caused by a low agitation speed of 35 rpm resulted in heterogeneous cell adhesion on Cytodex3. Additionally, on day 1 after seeding, the cell adhesion ratio reached 86.47% (Figure 1B), and then the cells embarked on the logarithmic growth with the maximum cell density up to 7.54 × 10^5^ cells/mL on days 5–6. Then, the cell density decreases from day 6, which could be ascribed to the fast consumption of nutrients in the batch culture. Interestingly, we found the cell adhesion ratio (on day 1) reached 98.68% with a shorter induction period upon the rotating speed increased to 45 rpm (Figure 1C). The cell keeps excellent viability in the whole process (100% within 5 days, Figure 1D). In addition, on the third day of inoculation, the cells grow uniformly on the microcarrier. Additionally, on day 5, the microcarrier agglomerates and the cell density reach the maximum value of 8.76 ± 0.19 × 10^5^ cells/mL, superior to that at an agitation speed of 35 rpm (7.54 × 10^5^ cells/mL). Unfortunately, upon the speed being set to 55 rpm, the induction period was prolonged to about two days, and the cell adhesion rate was only 51.32%, with the declined maximum cell density (5.02 × 10^5^ cells/mL, Table 1), indicating that the cells were completely intolerant to a higher shear force.

**Figure 2:**
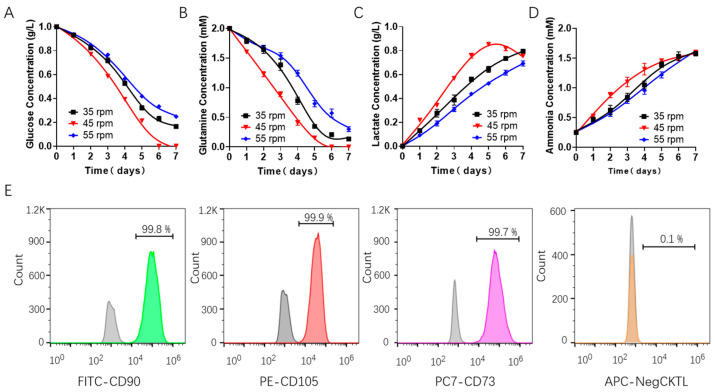
Cell metabolism characterization of batch-cultured hUCMSCs in 200 mL spinner flask. metabolite concentration assessment in the medium, including (**A**) glucose, (**B**) glutamine, (**C**) lactate and (**D**) ammonia. (**E**) Characterization of the hUCMSCs surface markers by flow cytometry using positive markers (FITC CD90, PE CD105 and CD73) and negative ones (APC CD14, CD19, CD34, CD45 and HLA-DR), where the gray and colored peak respectively represent the control and experimental group.

Then, we evaluated the effects of shear force on the consumption of nutrients (including glucose and glutamine) and the production of metabolic waste such as lactic acid and ammonia during the batch culture in a spinner flask using Roche biochemical analysis. The results showed that the concentrations of glucose and glutamine in the hUCMSCs medium (Figure 2A,B) decreased steadily during the culture. Obvious differences in the consumption of nutrients were observed under different rotating speeds. The concentrations of lactic acid and ammonia in the hUCMSCs medium (Figure 2C,D) also increased steadily. Surprisingly, we found the cells began to consume lactic acid for growth when the rotating speeds were set to 45 rpm. Importantly, the toxicity of lactic acid (1 g/L) and ammonia was not observed at all the three speeds, indicating that the accumulation of waste did not directly affect the growth of hUCMSCs [37,38].

We also evaluated the characteristic surface markers of the stem cells cultured at 45 rpm and found that hUCMSCs highly expressed positive CD90, CD105 and CD73 markers, with an expression level of more than 99%, and did not express negative markers such as CD14, CD19, CD34, CD45 and CD HLA-DR, which met the criteria of hUCMSCs.

According to the aforementioned results, the fluid shear force corresponding to a rotating speed of 45 rpm/min in the spinner flask seems more favorable for stem cell scale-up expansion, in which the hUCMSCs exhibited the highest cell adhesion ratio and viability as well as the inheritance of stem cell phenotype.

### 3.2. CFD Model for Scale-Up Criteria of hUCMSCs from 200 mL Spinner Flask to BioStar 1.5c

**Figure 3:**
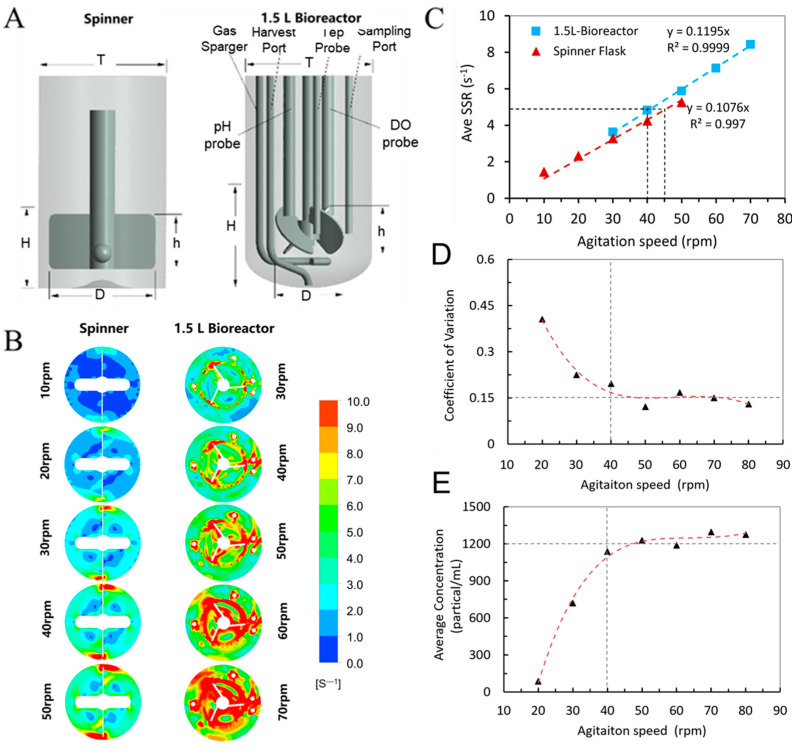
(**A**) Geometry of the spinner flask and BioStar 1.5c. (**B**) Horizontal slice contour plots of the shear strain rate in the spinner flask and BioStar 1.5c. (**C**) Volume-averaged shear strain rate relationship between the BioStar 1.5c and spinner flask to determine an appropriate scale-up value. (**D**,**E**) Coefficient of variation and average concentration of the microcarrier density in BioStar 1.5c, the triangles mean a value calculated by CFD andthe dashed line represent the fitted curve.

The evaluation of cell growth kinetics and nutrient metabolism in a spinner flask provides theoretical support for the three-dimensional expansion culture of hUCMSCs in a larger bioreactor. Especially, a rotary speed of 45 rpm in the spinner flask means a favorable fluid dynamic environment. Based on the cultivation process of hUCMSCs in spinner flasks, we focused on establishing a criterion to scale up the hUCMSCs to a 1.5 L tank bioreactor (BioStar 1.5c, Figure 3A). The main challenges resulted from the significant difference in tank geometry and stirred system between the two vessels. For example, the spinner flask has a flat bottom and is equipped with a large paddle impeller (D = 50 mm), while the BioStar 1.5c has an elliptical bottom and is agitated by an overhead motor and a 45° elephant ear impeller. Fortunately, utilizing CFD modeling, the geometry differences and impeller configurations between small and large-scale vessels were comprehensively considered in the modeling. In this study, the hydrodynamics of stirred tank bioreactors were investigated at two different scales (200 mL spinner flask and BioStar 1.5c, work volume was, respectively, 100 and 600 mL). The spinner flask was used as a baseline for the scale-up expansion of hUCMSCs to the 1.5 L bioreactor. A total of 10 models were run by testing the two different vessels at five different speeds (10, 20, 30, 40 and 50 rpm for the flask and 30, 40, 50, 60 and 70 rpm for Bioreactor 1.5c). Herein, we mainly focus on the shear strain rate (SSR) distribution, solved by commercial software ANSYS CFX in every grid within the bioreactors. The generated horizontal slice contour plots (Figure 3B) revealed that the highest values were located at any walls in the system, that is, the impeller tips and probes in BioStar 1.5c. The bulk liquid volume had a relatively low SSR at low agitation rates in both vessels. However, as the agitation rate increased, there was also an increased SSR in the bulk media, especially within the rotating domain, with peak values occurring at the tips of the impeller. At the completion of the simulations, postprocessing was performed to determine the volume-averaged SSR and the suspension performance of microcarriers within the spinner flask and BioStar 1.5c. It was shown that the averaged SSR has a linear correlation with agitation speed (Figure 3C), which is consistent with the previous study [39]. As the agitation rate increased, there was also an increase in the SSR in the bioreactor, and the correlation coefficient exceeded 0.99. Due to the differences in geometry, it was observed that the slopes of the SSR fit curves between the two vessels are quite different. Despite this, based on the two generated trend lines, we were able to predict the favorable agitation speed for hUCMSCs expansion in BioStar 1.5c by matching the volume-averaged SSR as seen in the spinner flask. For example, based on this correlation, the expansion agitation speed of 45 rpm in the spinner flask corresponds to 40 rpm in the 1.5 L bioreactor. Additionally, it is compulsory to ensure the suspension of microcarriers for the hUCMSCs cultivation. The suspension performance of the microcarriers was estimated based on the test of microcarrier density distribution in different locations. As shown in Figure 3D,E, the coefficient of variation and average concentration of the microcarrier density in different locations reached a steady value as the agitation speed exceeded 40 rpm, revealing the microcarriers were totally suspended. Based on the consideration of similar SSR and ensuring the suspension performance of microcarriers, the agitation speed of 40 rpm was used for the stem cell expansion in the 1.5 L bioreactor.

### 3.3. Scale-Up Expansion of hUCMSCs in BioStar 1.5c by Batch Culture

**Figure 4:**
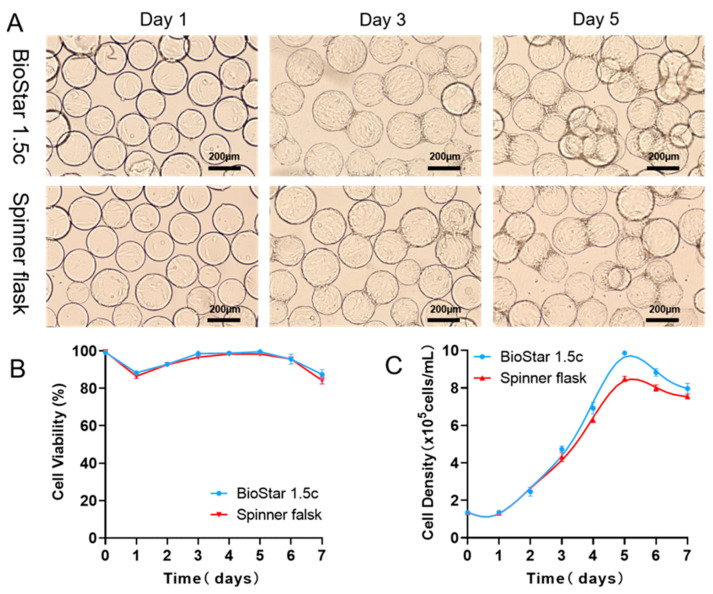
(**A**) Morphology and distribution of batch-cultured hUCMSCs on microcarriers at different time points (Day 1 to Day 7) cultured in both BioStar 1.5c and the spinner flask. (**B**,**C**) Cell viability and density during expansion of hUCMSCs. Data expressed as the mean ± standard deviation (*n* = 4 per group, per time point).

Inspired by the CFD simulation results, we concluded that the fluid force field corresponding to an agitation speed of 40 rpm is probably more favorable for hUCMSCs expansion in BioStar 1.5c. To verify this assumption, hUCMSCs were inoculated into BioStar 1.5c with a density of 1.35 × 10^5^ cells/mL (work volume, 600 mL), and the cells cultured in a spinner flask were used as a control. As shown in Figure 4, after 24 h of incubation, the cells could adhere to the microcarriers and keep proliferation with an agitation speed of 40 rpm. On day 5, all the microcarriers were homogeneously occupied by the cells. Adhesion rates and maximum density were, respectively, calculated as 98.36 ± 1.02% and 9.86 ± 1.35 × 10^5^ cells/mL (Table 2, speed: 40 rpm), which is higher than other speeds and close to that in a 200 mL spinner flask (98.13 ± 1.24% and 8.45 ± 1.15 × 10^5^ cells/mL). All of these indicated that computational fluid dynamics (CFD) simulation could act as a novel scale-up strategy to effectively expand the hUCMSCs in a 3D tank bioreactor, where the geometry differences between small and large-scale vessels could be comprehensively considered to predict the favorable flow field characteristics in different size and/or vessels shape. In addition, the cell viability reached 86%, and there was no difference between the two culture systems (Figure 4B). Notably, on the third day of culture, the hUCMSCs in BioStar 1.5c with the optimized speed (40 rpm) were more uniform than those in the spinner flask. On day 5, the cells cultured in BioStar 1.5c gradually increased up to 9.86 ± 1.35 × 10^5^ cells/mL (Figure 4C), which was significantly different from the hUCMSCs cultured in the spinner flask (8.45 ± 1.15 × 10^5^ cells/mL). The advantages of BioStar 1.5c could be ascribed to the computer-controlled dissolved oxygen (40%) and pH (7.2) during the culture process, which maintained an excellent physical environment for cell proliferation. The results also indicated that the pH and dissolved oxygen of the culture environment played a role in the growth of hUCMSCs, independent of the culture scale. In addition, during the whole culture process, the cellular morphological and growth kinetics characteristics (viability and density) are consistent with those in the spinner flask, indicating the feasibility of hUCMSCs expansion from 200 mL spinner flask to 1.5 L bioreactors by matching similar shear environment and suspending the microcarrier enabled by CFD simulation strategy.

**Figure 5:**
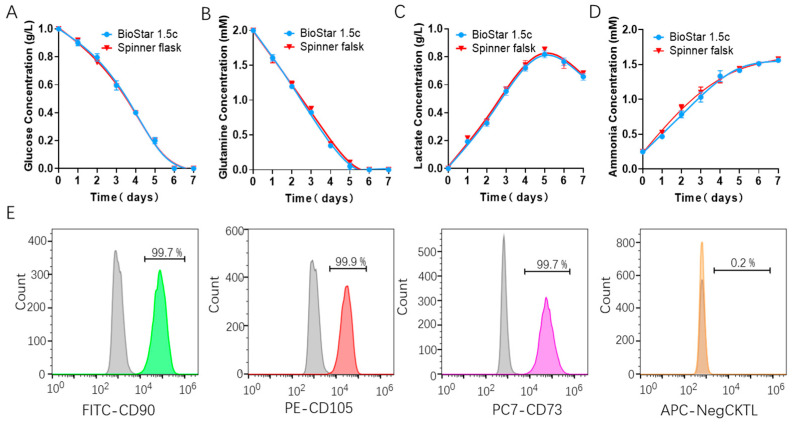
Metabolite concentration assessment of the batch-cultured hUCMSCs medium from BioStar 1.5c and the spinner flask, including glucose (**A**), glutamine (**B**), lactate (**C**) and ammonia (**D**). (**E**) Characterization of the hUCMSCs surface markers by flow cytometry using positive markers (FITC CD90, PE CD105 and CD73) and negative ones (APC CD14, CD19, CD34, CD45 and HLA-DR), where the gray and colored peak respectively represent the control and experimental group.

Similarly, nutrient (glucose and glutamine) consumption and metabolic waste production in BioStar 1.5c under the simulated rotary speed of 40 rpm were measured to check the metabolic characteristics of the batch-cultured hUCMSCs. The results showed that both the glucose and glutamine in the medium (Figure 5A,B) had a decreasing concentration, and there is no obvious difference between BioStar 1.5c and the spinner flask. In addition, the concentration of lactic acid and ammonia in the hUCMSCs medium (Figure 5C,D) had the same change trend in the two vessels. For example, both the lactic acid decreased after day 5 with an increasing ammonia level. Moreover, the toxicity of lactic acid (1 g/L) and ammonia (1.6 mmol/L) was not observed in the rotating flask or BioStar 1.5c [38,40]. In addition, the hUCMSCs cultured in BioStar 1.5c shared a consistent metabolic dynamic with that in the spinner flask. We further analyzed the quality of the expanded hUCMSCs in BioStar 1.5c. It was found that the positive CD90, CD105 and CD73 markers were highly expressed (up to 99%) in hUCMSCs. While the negative markers such as CD14, CD19, CD34, CD45 and CD HLA-DR were not expressed, meeting the criteria of stem cell culture and identification.

### 3.4. Scale-Up Expansion of hUCMSCs by Fed-Batch Culture

**Figure 6:**
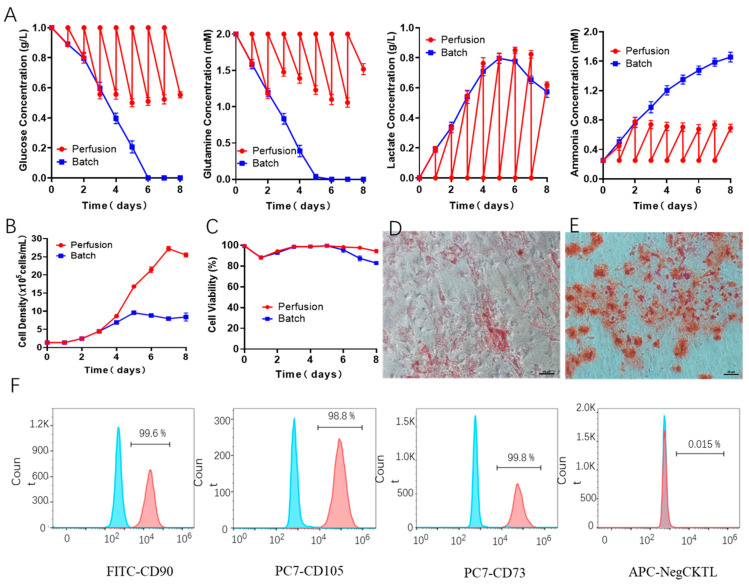
(**A**) Metabolite concentration assessment of the hUCMSCs medium from BioStar 1.5c when batch or fed-batch culture, including glucose, glutamine, lactate and ammonia. (**B**,**C**) Cell density and viability. Adipogenic (**D**) and osteogenic (**E**) differentiation evaluation of the expanded hUCMSCs by fed-batch culture in BioStar 1.5c. Surface markers characterization of the expanded hUCMSCs by fed-batch culture in BioStar 1.5c by flow cytometry (**F**), where the gray and colored peak respectively represent the control and experimental group.

As known to us, for the aforementioned batch culture, the consumption of nutrients in a closed system will affect the growth kinetics and metabolic characteristics of hUCMSCs. Especially at the end of the expansion, the cells faced limited growth and even cell death, which significantly challenges the high-quantity and high-quality hUCMSCs for clinical therapy. In order to further optimize the three-dimensional culture of hUCMSCs in BioStar 1.5c (agitation speed: 40 rpm) and extend the cell number and physiological environments of hUCMSCs, we used a fed-batch culture strategy, and the medium was refreshed every 24 h. As shown in Figure 6A, across the whole process, the glucose and glutamine concentration in the medium was consecutively maintained at above 0.5 g/L and 1 mM. As a comparison, the glucose and glutamine were completely consumed on day 5 for batch culture. In addition, the lactic acid and ammonia reached the maximum concentration of 0.8 g/L and 0.82 mM. It can be seen that this fed-batch strategy can effectively control the concentration of lactic acid in the culture medium. As a result, the hUCMSCs in our BioStar 1.5c continued proliferation within the 7 days upon fed-batch culture, and the maximum cell density reached up to 27.3 × 10^5^ cells/mL on day 7. While for the batch culture, the cells stopped proliferating on day 5 and followed a declined cell density and viability (Figure 6B,C). Notably, the maximum cell density of fed-batch culture (7.3 × 10^5^ cells/mL on day 7) is 2.9 folds of that of batch culture, which could achieve 20.2 times expansion (compared to seeded cells), significantly higher than 2D plates culture (12.0 × 10^5^ cells/mL, 8.9 folds) and most of the reported work [41,42,43,44,45].

Compared to other MSCs such as human bone marrow MSCs, hUCMSCs exhibited less donor variation [46,47], which is more favorable for MSCs scale-up ampliation. Even so, it is still challenging to ensure that the majority of seeding MSCs homogeneously grown in BioStar 1.5c under the parameters described here. To learn the homogeneity of amplified MSCs, differentiation potential and characteristic surface markers were comprehensively evaluated. As shown in Figure 6D–F, the expanded hUCMSCs by fed-batch culture in BioStar 1.5c maintained the adipogenic and osteogenic differentiation potential and still expressed the characteristic surface markers of hUCMSCs in a high population (>98%), indicating the ignorable MSCs variation. The whole amplification process provides guidance for the quality standardization and yield controllability by CFD simulation, which has been proved as an efficient toolbox for promoting the hUCMSCs industrial expansion for clinical therapy by matching the shear environment in different bioreactors.

## 4. Conclusions

A novel strategy using the computational fluid dynamics (CFD) model was developed to effectively scale up the hUCMSCs manufacturing processes in 3D bioreactors. We successfully translated and expanded the hUCMSCs from a 200 mL spinner flask to a 1.5 L computer-controlled bioreactor by matching the shear environment and suspending the microcarrier. Experimental results revealed that the batch-cultured hUCMSCs in bioreactors with an agitation speed of 40 rpm shared a more favorable growth and metabolism dynamic, similar to that in spinner flask (45 rpm), showing comparability in both systems. Notably, the maximum cell density reached up to 27.3 × 10^5^ cells/mL by fed-batch culture, 2.9-folds of that of batch culture and 20.2 times of seeding cells. In this article, efficient process optimization and scale-up expansion of hUCMSCs were achieved in a bioreactor system by CFD simulation strategy, which would provide an alternative toolbox to generate massive and standardized curative cells and open up a broader experimental research prospect for clinical therapy.

## Figures and Tables

**Table 1:** Effects of agitation speeds on the proliferation of hUCMSCs cultured in 200 mL spinner flask.

Speeds (rpm)	Inoculum	Adherence Rate (Day 1)	Maximum Density	Maximum Specific Growth Rate (μ_max_)
	(×10^5^ cells/mL)	(%)	(×10^5^ cells/mL)	(h^−1^)
35	1.35	86.47 ± 1.26	7.54 ± 0.27	0.0262 ± 0.0022
45	1.35	98.68 ± 0.15	8.76 ± 0.19	0.0268 ± 0.0014
55	1.35	51. 32 ± 2.48	5.02 ± 1.88	0.0249 ± 0.0010

**Table 2:** Effects of the two culture methods on the proliferation of hUCMSCs.

Culture Methods	Speeds (rpm)	Inoculum (×10^5^ cells/mL)	Adherence Rate (%)	Maximum Density (×10^5^ cells/mL)	Maximum Specific Growth Rate (μmax) (h^−1^)
BioStar 1.5c	30	1.35	78.56± 2.65	8.13 ± 0.31	0.02554 ± 0.026
	40	1.35	98.36 ± 1.02	9.86 ± 1.35	0.02722 ± 0.030
	50	1.35	81.36 ± 1.23	7.59 ± 0.26	0.02561 ± 0.025
	60	1.35	65.21 ± 2.23	5.68 ± 0.16	0.0240 ± 0.012
	70	1.35	45.62 ± 1.45	4.35 ± 0.89	0.02381 ± 0.056
Spinner flask	45	1.35	98.13 ± 1.24	8.45 ± 1.15	0.02719 ± 0.042

## Data Availability

Data are available by request to the corresponding author.
